# Differences in Gene Transcriptomic Pattern of *Plasmodium falciparum* in Children with Cerebral Malaria and Asymptomatic Carriers

**DOI:** 10.1371/journal.pone.0114401

**Published:** 2014-12-05

**Authors:** Talleh Almelli, Grégory Nuel, Emmanuel Bischoff, Agnès Aubouy, Mohamed Elati, Christian William Wang, Marie-Agnès Dillies, Jean-Yves Coppée, Georges Nko Ayissi, Leonardo Kishi Basco, Christophe Rogier, Nicaise Tuikue Ndam, Philippe Deloron, Rachida Tahar

**Affiliations:** 1 Institut de Recherche pour le Développement (IRD), UMR 216 Mère et Enfant Face aux Infections Tropicales, Université Paris-Descartes, Près Sorbonne Paris-Cité, Paris, France; 2 PRES Sorbone Paris Cité, Université Paris Descartes, Faculté de Pharmacie, Paris, France; 3 Institut Pasteur, Unit of Molecular Immunology of Parasites, Unit of Insect Vector Genetics and Genomics, Department of Parasitology and Mycology, Paris, France; 4 Centre National de la Recherche Scientifique (CNRS), URA 3012, Paris, France; 5 Institut de Recherche pour le Développement (IRD), UMR 152 Pharmacochimie et pharmacologie pour le développement - (PHARMA-DEV), Université Paul Sabatier, Toulouse, France; 6 Institute of Systems and Synthetic Biology, CNRS, University of Evry, Genopole, Evry, France; 7 Centre for Medical Parasitology at Department of International Health, Immunology, and Microbiology, University of Copenhagen and at Department of Infectious Diseases, Copenhagen University Hospital (Rigshospitalet), Copenhagen, Denmark; 8 Plate-forme Transcriptome et Epigénome, Departement Génomes et Génétique, Institut Pasteur, Paris, France; 9 Hôpital d′Olembé, Yaoundé, Cameroon; 10 Organisation de Coordination pour la lutte contre les Endémies en Afrique Centrale (OCEAC), Laboratoire de Recherche sur le Paludisme, B. P. 288, Yaoundé, Cameroon; 11 Institut de Recherche pour le Développement (IRD), UMR 198 Unité de Recherche des Maladies Infectieuses et Tropicales Emergentes, Faculté de Médecine La Timone, Aix-Marseille Université, Marseille, France; 12 Institut Pasteur de Madagascar, B.P. 1274, Ambatofotsikely, Antananarivo, Madagascar; London School of Hygiene and Tropical Medicine, United Kingdom

## Abstract

The mechanisms underlying the heterogeneity of clinical malaria remain largely unknown. We hypothesized that differential gene expression contributes to phenotypic variation of parasites which results in a specific interaction with the host, leading to different clinical features of malaria. In this study, we analyzed the transcriptomes of isolates obtained from asymptomatic carriers and patients with uncomplicated or cerebral malaria. We also investigated the transcriptomes of 3D7 clone and 3D7-Lib that expresses severe malaria associated-variant surface antigen. Our findings revealed a specific up-regulation of genes involved in pathogenesis, adhesion to host cell, and erythrocyte aggregation in parasites from patients with cerebral malaria and 3D7-Lib, compared to parasites from asymptomatic carriers and 3D7, respectively. However, we did not find any significant difference between the transcriptomes of parasites from cerebral malaria and uncomplicated malaria, suggesting similar transcriptomic pattern in these two parasite populations. The difference between isolates from asymptomatic children and cerebral malaria concerned genes coding for exported proteins, Maurer's cleft proteins, transcriptional factor proteins, proteins implicated in protein transport, as well as *Plasmodium* conserved and hypothetical proteins. Interestingly, UPs A1, A2, A3 and UPs B1 of *var* genes were predominantly found in cerebral malaria-associated isolates and those containing architectural domains of DC4, DC5, DC13 and their neighboring *rif* genes in 3D7-lib. Therefore, more investigations are needed to analyze the effective role of these genes during malaria infection to provide with new knowledge on malaria pathology. In addition, concomitant regulation of genes within the chromosomal neighborhood suggests a common mechanism of gene regulation in *P. falciparum*.

## Background

Of the five malaria species that infect humans, *Plasmodium falciparum* is the most common in Africa, and is the species that causes the most severe disease. According to the World Health Organization (WHO), the majority (86%) of the estimated 660 000 annual deaths related to malaria are due to *P. falciparum* infections that occur in children under the age of five years old living in Africa [1]–[4]. Despite an adequate antimalarial treatment, 10 to 30% of patients with severe malaria die, and 25% of survivors of cerebral malaria develop chronic neuro-cognitive impairment [5]. Indeed, the dramatic loss of lives due to malaria and the negative economic impact associated with the disease lead to the loss of approximately 412 billion US dollars in African countries yearly, further retarding their economic development [6]. At present, the only means to effectively control malaria in human hosts remains the use of antimalarial drugs. However, malaria parasites are capable of developing resistance to all currently available drugs, and only a few promising novel drug candidates are under development [7]. Furthermore, despite several candidates undergoing early phases of clinical development, a highly effective vaccine against malaria parasites is still unavailable. The identification of new therapeutic targets or vaccine candidates is hampered by the lack of knowledge on various molecular mechanisms used by malaria parasites to survive within the human host.

*P. falciparum* infection causes a large spectrum of disease manifestations, ranging from asymptomatic infection (AM) to uncomplicated malaria (UM) and severe and complicated malaria. Only a small proportion of individuals develops severe disease, including cerebral malaria (CM), severe malaria-associated anemia (SMA), and other life-threatening clinical syndromes, such as acute renal failure, acute respiratory distress, pulmonary edema, hemoglobinuria, disseminated intravascular coagulation, and circulatory collapse [8], [9]. In areas of low transmission, severe malaria also occurs in older children (i.e.> 5 years old) and adults. After repeated malaria episodes, individuals develop natural protective immunity and considerably decrease their risk to develop severe and complicated malaria, suggesting acquired immunity and protection against severe disease [10]–[12]. The heterogeneity of clinical manifestations is due to both human and parasite factors [13]–[15].

Although the biological processes involved in the progression from acute uncomplicated malaria towards severe clinical state are mostly unknown, parasite gene expression changes depending on the clinical manifestations may occur in genes involved in virulence.

The relationship between parasite genetic profiles and malaria-related pathologies needs to be established to further understand the pathogenesis of severe and complicated malaria.

Genomic research on *P. falciparum* has advanced considerebly since the entire genome sequence of the *P. falciparum* 3D7 reference clone was determined. *P. falciparum* 23-Mb genome is AT-rich and comprises 14 nuclear chromosomes, a 6-kb mitochondrial genome, and a 35-kb plastid-like apicoplast genome [16]. The genome sequencing project revealed a large proportion of predicted genes which are likely to be involved in parasite evasion mechanisms from human immune system, and host-parasite interactions. The majority of these latter genes encode amplified gene families, such as the hypervariable variant surface antigen (VSA) genes (*var*, *rif*, *stevor*, and *pfmc2TM*) that mediate evasion from the host immune system, and genes involved in host cell invasion pathways. The achievement of gene transcription profiling of the intraerythrocytic developmental cell cycle, proteomic studies, and *in silico* sequence computational analysis have led to a substantial progress in defining gene functions and genetic regulatory networks [17]–[20]. However, the absence of data from a large number of *P. falciparum* field isolates associated with different clinical manifestations, ranging from asymptomatic carriage to uncomplicated malaria and severe disease, tends to limit our knowledge on the biological processes involved in parasite virulence.

It has been shown that parasites isolated from children with severe malaria (SM) express a limited and conserved set of VSA antigens (VSA-SM) that are more strongly and more commonly recognized by IgG from malaria-exposed individuals than VSA expressed by parasites infecting children with uncomplicated malaria (VSA-UM) [21]. Therefore, the identification of VSA-SM encoding genes may reveal new therapeutic and vaccine targets for malaria control.

The aim of this study was to analyze and compare the transcriptomes of *P. falciparum* parasites isolates from asymptomatic carriers and patients with clinical malaria, including UM and CM, by DNA microarray hybridization, in order to identify potential virulence factors associated with severe clinical manifestations. As controls for our experiments, we analyzed and compared the transcriptome of 3D7 reference clone to that of 3D7 Lib that expresses VSA-SM related to severe pathogenesis [21], [22] and differentially transcribes a set of *var* and *rif* genes.

## Patients, Materials and Methods

### Patients

After obtaining verbal informed consent from the children's parents or guardians, Cameroonian schoolchildren aged <12 years old were mass-screened in November 2009 to identify asymptomatic *P. falciparum* malaria parasite carriers (AM) in Ekoundouma, a village located about 5 km west of Yaoundé (Latitude:3.8644652°, Longitude:11.5138617°). Thick smears prepared from fingerpricked capillary blood samples were stained with 10% Giemsa, and the parasite density of *P. falciparum* was determined by microscopy. Children with positive thick blood smear who had not taken antimalarial treatment within the previous two weeks and who were afebrile at the time of mass screening and during the previous one week were enrolled after obtaining verbal informed consent from their parents or their caretakers. Children with gametocytemia, mixed infections with *P. malariae*, or fever (axillary temperature > 37.5°C) were excluded. Schoolchildren with >1,000 asexual parasites/µL of blood and symptoms associated with malaria were treated with artesunate-amodiaquine combination and paracetamol, as recommended by the Cameroonian Ministry of Public Health.

Symptomatic patients with acute uncomplicated malaria (UM) were enrolled among patients consulting spontaneously at the Nlongkak Catholic missionary dispensary in Yaoundé from January to December 2009. The inclusion criteria were as follows: age <12 years old, *P. falciparum* parasitemia ≧ 0.1% (or > 5,000 asexual parasites/µL of blood), absence of other *Plasmodium* species, fever (axillary temperature > 37.5°C) at the time of consultation or history of fever within 24 h preceding consultation, absence of signs and symptoms of severe and complicated malaria, as defined by the WHO, and denial of recent self-medication with an antimalarial drug. The enrolled patients were treated with artesunate-amodiaquine and paracetamol.

Children presenting with cerebral malaria (CM) were enrolled at Olembe hospital in Yaoundé, Cameroon, between April 2008 and December 2009, and at the Centre National Hospitalier Universitaire Hubert Koutoucou Mega (CNHU-HKM) in Cotonou, Benin, in 2012 and 2013 between June and August. CM was defined as a Blantyre coma score of ≤ 2 persisting for 30 min and/or at least two seizures within 24 h preceding consultation, and no other obvious cause of coma. The patients were given the appropriate treatment and necessary medical care at the hospital, according to the current guidelines for the treatment of severe and complicated malaria recommended by the Cameroonian and the Beninese Ministries of Health.

Clinical history, physical and neurological examinations, as well as laboratory examinations, including *P. falciparum* parasitemia, creatinine, glycemia, and C-reactive protein, were registered on an *ad-hoc* data form.

After written informed consent was obtained from symptomatic children's parents or legal guardian, *P. falciparum*-infected blood was collected by venipuncture. Approximately 3–5 mL of blood was collected in ethylene diamine tetraacetic acid (EDTA)-coated tubes. The study protocol was reviewed and approved by the Cameroonian National Ethics Committee and the Cameroonian Ministry of Public Health (authorization No 028/CNE/DNM/07) as well as by the ethics committee of the Research Institute of Applied Biomedical Sciences, Cotonou, Benin (No 006/CER/ISBA/12 and N°21/CER/ISBA/13).

We obtained verbal informed consent from all parents or guardiants of schoolchildren who participated in the current study, including those from whom venous blood was not collected.

For all symptomatic children enrolled in the study, written informed consent was obtained from their parents or legal guardians.

Verbal informed consent complied with the national ethics committee rules and recommendations at the time the study was performed.

The written informed consent form and its recording procedures were approved by the two ethics committees.

### Cultivation of *P. falciparum* parasites

Two laboratory-adapted parasite lines were used. Cryopreserved stocks of 3D7 reference clone and 3D7-Lib line derived from 3D7 [22] were cultivated for 10 days using the standard method developed by Trager and Jensen [23]. Parasites were synchronized, and ring and late trophozoite stages were isolated using plasmagel [24]. The 3D7-Lib line was obtained by several rounds of incubation with Liberian hyper-immune plasma using anti-IgG coupled with streptavidine-conjugated Dynabeads, as previously described [21]. The 3D7-Lib line was shown to express group A *var* and neighboring *rif* genes, suggesting that it mainly expresses variable surface antigens (VSAs) associated with severe disease [25]–[27].

### Sample collection

A total of 214 blood samples were collected (111 from asyptomatic children, 70 from children with uncomplicated malaria, and 33 from children with cerebral malaria). Three samples contained circulating late trophozoites, one from a patient with uncomplicated malaria and 2 from asymptomatic carriers. Of 111 samples obtained from asymptomatic children, 6 were excluded due to mixed infections with *P. malariae* and 11 were excluded due to the presence of circulating *P. falciparum* gametocytes. Forty-four of 61 (72%) UM samples, 20 of 33 (59%) CM samples and 31 of 48 (64%) isolates from asymptomatic carriers grew successfully to late trophozoite stage.

### Maturation of Plasmodium falciparum isolates

Venous samples containing late trophozoites were excluded, and only samples with 100% ring stage parasites were used in the present study. Samples were washed in cold phosphate-buffered saline (PBS) by centrifugation and divided into two fractions of 200 µL cell pellets. One fraction was immediately stored at −80°C in Trizol (Life Technologies) for RNA extraction. The second aliquot was cultivated for 24 to 38 h to obtain late trophozoites. Isolates that developed into at least 85% of late trophozoite stages were harvested and stored at −80°C in Trizol.

### Microarray design and hybridization

For each clinical group, three samples were constituted by pooling equivalent amounts of parasite RNA from six isolates to perform array hybridization. To ensure that the same quantity of RNA from each isolate was analyzed, we used isolates of approximately the same parasitemia. For the controls (3D7 clone and 3D7-Lib line), three samples (at either ring or late trophozoite stage) were obtained from three independent parasite cultures.

Microarrays were manufactured by Agilent Technologies using SurePrint *in situ* synthesis technology (Agilent Technologies France SAS, Les Ulis, France). Fifteen slides of eight arrays each containing 14,882 oligonucleotides covering 5,534 Open Reading Frame (ORF) sequences derived from the *P. falciparum* database were used. The oligonucleotides were designed using oligoarray2 software [28] by setting the general parameters to 55–60 bases for oligonucleotides length with a melting temperature between 79 and 81°C, and with a 3′ bias of 1,500 nucleotides since RNases preferentially act from the 5′ end. The melting temperature range was relaxed for genes for which no oligonucleotide could be found with the default parameters. The array contained two sets of oligonucleotides. The first set was designed on the basis of the 3D7 sequence genome. These oligonucleotides were chosen within the coding sequences (CDS) of protein-coding genes in the nuclear genome. A minimum of three oligonucleotides per gene were designed when possible. To avoid cross-hybridization with human genes, cross-hybridization was computed using both 3D7 and human genome libraries. The second set of oligonucleotides was designed to hybridize to genes exhibiting large sequence variations, including *var, rif, stevor* and *Pfmc-2TM* multigenic families. For these genes, all sequences available in the GenBank and those kindly provided by our collaborators (T. Lavstsen, Centre for Medical Parasitology, University of Copenhagen, Denmark) were retrieved and added to the array. Sequence alignment using Clustal W and Clustal X version 2.0 [29] was performed for each family or sub-family, and specific oligonucleotides were designed. For the other genes with high sequence variations (i.e., genes with high numbers of single nucleotide polymorphisms [SNPs]), sequences were retrieved from PlasmoDB, and specific oligonucleotides were designed.

Total RNA from 200 µl of *P. falciparum*-infected cell pellet and laboratory-adapted parasites (3D7 and 3D7-Lib) was extracted using chloroform-isopropanol method as recommended by the manufacturer. The integrity of the total RNA from each sample was checked using Agilent 2100 Bioanalyser (Agilent Technologies, France). For reverse transcriptase (RT)-real time PCR (rtPCR), RNA was treated with DNAse at 37°C for 15 min. The absence of contaminating DNA was assessed by 40 cycles of rtPCR using primers targeting *seryl tRNA transferase* and *fructose biphosphate aldolase* genes, which are highly conserved *P. falciparum* housekeeping genes.

Total RNA (6–10 µg) was transcribed using the SuperScript First-Strand Synthesis System (Life Technologies) and a mixture of random hexamer oligonucleotides. The newly synthesized cDNA was labeled using SuperScript indirect cDNA labeling system (Invitrogen, Carlsbad, CA) to generate green fluorescent Cyanin-3 (Cy3) labeled cDNA according to the manufacturer's instructions. The quantity of labeled cDNA was measured using Nanodrop (Thermo Fisher Scientific, Villebon-sur-Yvette, France) to ensure that the same quantity of labeled cDNA was deposited for each array hybridization.

After quantification of the fluorescent specific activity of Cy3-labeled cDNA, the quantities of targets were normalized at 100 pmol of Cy3, and hybridized for 24 h at 65°C. The slide was washed with water and dried at room temperature. Slides were scanned using GenePix 4000 A scanner (Axon), and the images were analyzed using GenePix5 software (Axon). Dusted and poorly fluorescent spots (below the background level) were excluded from analysis. After array gridding, data were exported as gpr files for statistical analysis.

### Quantitative rtPCR

Quantitative rtPCR was performed to confirm array results. For each target gene, a pair of primers was designed to obtain specific DNA sequence fragments that span less than 350 bp. Primer sequences are presented in Table S1. Individual real-time rtPCR amplifications were carried out in a final volume of 20 µl in a Frame Star 384-well plate (4titude France, Bagneux, France) containing 1× final concentration of SYBR Green JumpStart *Taq* ReadyMix (Sigma-Aldrich, Saint-Quentin Fallavier, France),

100 nM gene-specific primers, and 1 µl cDNA. rtPCR was performed using 7900 HT (Applied Biosystems, Saint Aubin, France). The thermal cycle program was as follows: 10 minutes of pre-incubation at 95°C followed by 95°C × 15 sec and one min at 60°C for 40 cycles. All samples were run in triplicate. Threshold cycles (Ct) and melting curves were analyzed using Applied Biosystems ABI SDS software 2.3. The level of amplification referred to the number of cycles at which the PCR product was detectable and confirmed by the melting curve for each gene. Normalized data were used to quantify the relative levels of a given mRNA between samples using the ΔΔCt analysis [30]. Before proceeding to the relative quantification using this method, the similarity in PCR amplification efficiency of the target gene and a house keeping gene (argenyl tRNA synthetase, PF3D7_1218600/PFL0900c) used as internal controls was checked.

To validate the array results by rt-qPCR, a set of 14 genes were chosen randomly from genes that were differentially expressed between the clinical groups and between 3D7 and 3D7-Lib laboratory-adapted parasites. These genes were grouped into three categories, depending on their fold change with the arrays: down-regulated genes defined as Fold change [FC] value <2 units of expression), moderately up-regulated genes (FC value, 2–7 units of expression), and highly up-regulated genes (FC value > 7 units of expression). To further confirm our findings, additional rt-qPCR was performed for a set of *var* gene family found to be up-regulated by arrays analysis using a new batch of parasite RNAs isolated from children with CM or UM. The transcript abundance of *var* genes was expressed as Transcript units (T_u_), defined as T_u_ =  2(5-ΔCt) [31].

### Statistical analysis

Data generated from the arrays were analyzed using the R software 2.14.1 R Core Team 2012 http://www.R-project.org/ [32] and Bioconductor limma package [33]. Since hybridizations included only one condition per array, the resulting one-color hybridization signal was first loaded in a limma-like RG structure with the red foreground signal set to 1.

#### Background signals (green signal/red signal)

Since the Agilent microarrays (which are usually used as two-color cDNA arrays) were used as single color arrays, the standard loess normalization method for two-color microarrays did not apply in our study. Thus, a between-array quantile normalization which is the standard method for one-color Affymetrix arrays was applied to all arrays.

Background signals were set to 0 for both colors, and the RG data structure was transformed into MA using the RG.MA function, with logarithmic ratios in log 2 (green signal/red signal). A quantile normalization between arrays was applied to the full data set [34]. After exclusion of flagged (invalid) and control spots, differentially expressed genes were extracted using linear models and the empirical Bayes method implemented in limma [35], followed by a Benjamini and Yekutieli (BY) *P*-value adjustment with error value set to 0.05 [36].

The mean fold changes of differential expression between pairs of conditions were computed and a cut-off value of 2 was applied. Transcripts were considered as differentially expressed when all their probes had a fold change higher than 2 and a p-value lower than 0.05. Correlation (Rho) between qPCR and array results was determined by the rank-based non-parametric Spearman's test, and the Kendall test was used to test the correlation between our results and the published transcriptomic data.

Data are available at EMBL-EBI ArrayExpress (https://www.ebi.ac.uk/arrayexpress/arrays/A-MEXP-2369) using the following username and password: Username: Reviewer_A-MEXP-2369, Password: dxxahhsu, Username: Reviewer_E-MTAB-2139, Password: utM2tpQP for array design and experiment respectively

### Bioinformatics analysis

Functional analysis of the differentially expressed genes was performed using the Gene Ontology (GO) GOstats package in Bioconductor with default settings [37] and Sanger GeneDB *P. falciparum* gene annotations. The supplementary file lists the top five most overrepresented GO terms in each set (Table S2).

Differentially expressed genes were clustered according to their fold change using the neighbor joining algorithm with Lance-Williams dissimilarity updates (hclust function from R 2.15.1) starting from euclidian distance on fold changes.

Stage-matching analysis was performed by Canonical Correlation Analysis (CCA) to search for potential correlation between the transcriptomic data of our samples at ring and late trophozoite stages and data of 3D7 obtained hourly during the 48-h intra-erythrocytic life cycle [18]. A multivariate statistical model was used to facilitate the study of linear interrelationships between two sets of variables and find a set of basis vectors for two multidimensional variables such that the projections of variables, called canonical correlation, onto these basis vectors are maximally correlated. Each CCA component is associated with the corresponding canonical correlation that characterizes the strength of dependency [38]. We used an R package, CCA, to develop numerical and graphical outputs [39].

## Results

### Patients characteristics

Blood samples from 54 patients were used in the study: 18 children with cerebral malaria (CM), 18 children with acute uncomplicated malaria (UM), and 18 children with asymptomatic malaria (AM). The clinical and biological characteristics of these patients are summarized in Table 1. The mean age differences between patients of the three clinical groups were statistically significant (*P* <0.05). At enrolment, the mean body temperatures were comparable between CM and UM groups (*P* > 0.05). As expected, the asymptomatic carriers were all afebrile, and their mean body temperature was significantly lower (*P* <0.05) than that of the other two clinical groups. The CM group had significantly lower hemoglobin values than the UM group (*P* <0.05). The geometric mean parasite densities of CM and UM groups were not significantly different (*P* > 0.05). Asymptomatic carriers had a significantly lower parasite density compared with that of other patient groups (*P* <0.05). These differences in parasite densities were corrected before performing the arrays. To avoid bias in the expression pattern of abundant transcripts due to high parasitemia, RNAs from highly parasitized CM and UM isolates were diluted to obtain similar concentrations of RNA as in AM isolates.

**Table 1:** Clinical and biological characteristics of *P. falciparum*-infected patients and asymptomatic carriers.

Characteristics	Cerebral malaria^1^	Uncomplicated malaria	Asymptomatic carriers
Number of enrolled patients	18	18	18
Age (mean ± SD) (range) (months)	15.3 ± 6.6 (6–25)	28.9 ± 15.9 (7–60)	65.3 ± 7.4 (48–72)
Sex ratio F/M (n)	0.8 (8/10)	1.0 (9/9)	1.25 (10/8)
Geometric mean parasitemia (95% confidence interval; range) (asexual parasites/µl of blood)	30,000 (9,550–94,000; 300–309,000)	50,100 (38,200–65,600; 12,800–109,000)	3,460 (2,400–5,000; 1,120–9,630)
Body temperature (mean ± SD) (°C)	39.0 ± 1.1	38.9 ± 0.8	36.8 ± 0.4
Hematocrit (mean ± SD) (%)	24.6 ± 3.7	29.9 ± 5.7	ND

In two cases, symptoms of cerebral malaria occurred concomitantly with acute respiratory distress or hemoglobinuria. ND, not determined.

1 mean Blantyre score, 2/5; blood glucose level (mean ± SD), 1.17 ± 0.29 g/L; creatinine (mean ± SD), 12.7 ± 33.7 mg/dL.

Gene expression pattern of field isolates

**Figure 1:**
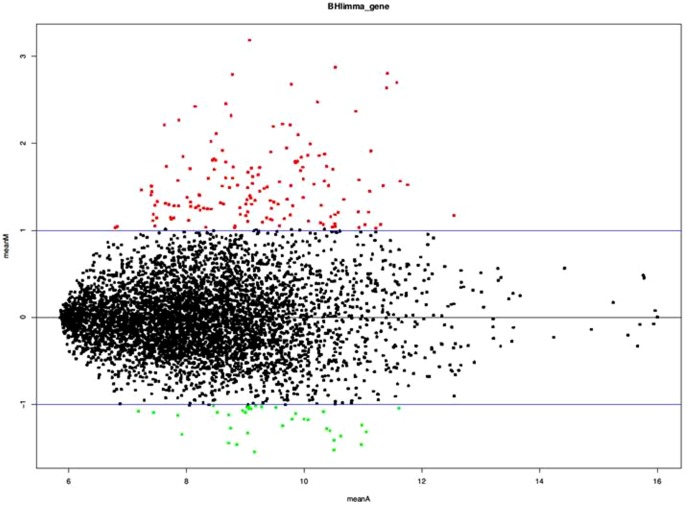
Mean MA plot associated with the comparison of cerebral malaria (CM) with asymptomatic malaria (AM) (mean log ratio of expression versus mean log intensity). Statistically significant data according to the Benjamini and Yekutieli P value correction are shown as red dots for up-regulated genes and green dots for down-regulated genes in CM-associated parasites.

For each clinical group, three pools of six isolates were prepared and hybridized on arrays at both ring and late trophozoite stages. These isolates were collected from children with CM, UM, or AM. The transcriptome of isolates after maturation was compared to that of late trophozoites of 3D7 reference clone. Furthermore, the transcriptome of clinical isolates from children with CM was also compared to that from children with AM and to that from children with UM. We also compared the transcriptome of the parent 3D7 clone to that of the selected 3D7-Lib. Figure 1 presents the MA plot of differentially expressed genes between these two conditions and Figure S1 presents the raw p-values obtained from limma for the comparison of cerebral malaria with asymptomatic malaria. Our transcriptomic data from 3D7 reference clone was also compared to those of the published database [40] which yielded a high correlation (r  =  0.>56, *P*  =  0.004) between the two transcriptomic data.

The transcriptome of the rings was obtained by hybridization of cDNA from a mixture of 80% rings, 5% late trophozoites, and 15% schizonts for laboratory-adapted parasite lines. All field isolates consisted of 100% ring stages. In parallel, the transcriptome of late trophozoites of both laboratory-adapted parasites and clinical isolates was analyzed by cDNA hybridization using a mixture of 85% late trophozoites, 10% schizonts, and 5% rings.

Using one-color labeling method, the mean green fluorescence intensity after removing the background signal reflects the presence or absence of cDNA corresponding to each gene among the 5,534 analyzed ORFs. The analysis of transcriptomic data of 3D7 and 3D7-Lib ring stage parasites provided 168 transcripts with a mean fluorescence level below the background, which were undetected. Likewise, in late trophozoite stages, 61 of these 168 transcripts were also not detected. However, the transcriptome data of field isolates at late trophozoite stage showed only 38 transcripts with fluorescence intensities below the background level. Transcriptomic data from 3D7 reference clone and field isolates at the same developmental stages were highly correlated (r  =  0.79) in most (4,837 to 5,344) transcribed ORFs. These results suggest that we detected the majority of isolate transcripts at both ring and late trophozoite stages. The lists of non-transcribed genes in 3D7, 3D7-Lib, CM-associated isolates, and AM-associated isolates are provided in supplementary information Table S2, S3, S4, respectively.

### Stage matching analysis results

CCA for stage-matching analysis showed that the transcriptome of our samples at the ring stage is positively correlated with the transcriptome of 3D7 between 1 and 8 hr and between 43 and 48 hr post-invasion. Similarly, the transcriptome of late trophozoite stage in our samples matches with that of 38 – 48 hr post-invasion parasites (Figure S3).

### Differentially expressed genes in 3D7-Lib and 3D7

3D7-Lib expresses VSAs that are potentially implicated in severe and complicated malaria, including cerebral malaria. The comparison of this selected 3D7-Lib to the parent 3D7 reference clone showed some differences in the expression profile. A total of 395 transcripts were up-regulated in 3D7-Lib late trophozoites, as compared to the corresponding developmental stage of 3D7. Of these 395 genes, 52 displayed 3- to 9-fold higher expression in 3D7-Lib. Some of these genes encode ring exported proteins REX1, REX2, REX3, erythrocyte membrane protein PfEMP1 members, membrane-associated histidine-rich protein (MAHRP1), histidine-rich protein II (HRPII), *Plasmodium* exported protein (PHISTa), (PHISTb, and PHISTc), DnaJ protein, putative serine/threonine protein kinase family (FIKK4.2), and hypothetical proteins. A total of 296 genes were slightly down-regulated, exhibiting 2- to 3-fold change in the expression level. The comparison of ring-stage transcriptomes revealed 30 up-regulated genes, ranging from 4- to 30-fold increased expression in 3D7-Lib, and five down-regulated genes. The majority of the overexpressed genes encoded proteins belonging to PfEMP1 and RIFIN families, exported proteins, knob-associated histidine-rich protein (KAHRP), proteins with DNAJ domain, and some hypothetical proteins. The group A *var* genes [PF3D7_1100200/PF11_0008, PF3D7_1150400/PF11_0521), PF3D7_1300300/PF13_0003), and PF3D7_0425800/PFD1235w], and neighboring *rif* genes [PF3D7_1100300/PF11_0009, PF3D7_1150300/PF11_0520, PF3D7_1300400/PF13_0004, and *rif* PF3D7_0425700/PFD1230c] were up-regulated in 3D7-Lib, as compared to 3D7, with an expression level ranging from 2.5 to 26-fold (Table S5). These genes were found to be up-regulated in 3D7-Lib in a previous study [22].

### Identification of differentially expressed genes in CM compared with AM

Only the comparison between late trophozoites from CM and AM patients showed statistically significant differentially expressed genes, leading to 99 up-regulated transcripts and 135 down-regulated transcripts (Table S5). However, the comparison between parasites from CM and UM patients showed no significant differences in the transcriptomic pattern.

The up-regulated genes in CM parasites compared to AM parasites exhibited 3 to 14 higher expression rates, while down-regulated genes showed 3- to 22-fold change. The 15 most up-regulated genes encode *Plasmodium* exported proteins, glycophorin-binding proteins (GBP), hypothetical proteins, VSA proteins, such as PfEMP1, RIFIN, other surface proteins like tryptophan- and threonine-rich antigen, and ring-infected erythrocyte surface antigen (RESA). Of the 99 up-regulated genes in CM-associated parasites compared to AM-associated parasites, 39% were also up-regulated in 3D7-Lib, and included members of *rif* A1 and *var* A group, some of them being topologically located in the neighborhood. In parallel, 135 transcripts were down-regulated in CM parasites, as compared to AM parasites. Among these 135 transcripts, 36 showed a 5- to 22-fold decrease in transcription and corresponded to genes encoding rhoptry-associated proteins (RAP1, RAP2, and RAP3), merozoite surface proteins (MSP3, MSP7, and MSP9), Maurer's cleft 2 transmembrane domain protein, and hypothetical proteins.

We then compared the transcriptome of CM-and AM-associated late trophozoite isolates to that of cognate laboratory-adapted 3D7. A significant difference in gene expression between field maturated trophozoites from individuals with CM and those with AM, compared to 3D7 was observed. In CM-associated isolates, 182 transcripts were up-regulated and 85 were down-regulated, while in AM-associated parasites 40 were up-regulated and 41 were down-regulated. The most up-regulated transcripts in CM parasites presented 2- to 9-fold changes, and the most down-regulated genes presented fold change ranging from 2 to 16 for the majority of transcripts. Most (50 to 67%) of the genes found to be transcribed in both CM-and AM-associated parasites compared to 3D7, belonged to *phist rifin*, *stevor*, *pfMc2-TM* and *pfemp1* encoding genes. In addition to VSA-encoding gene detection, we observed the up-regulation of genes encoding Skeleton Binding Protein1 (SBP1), HRPIII, glycophorin binding protein (GBPH2), as well as genes encoding *Plasmodium* conserved proteins for which no function has been assigned yet. Down-regulated genes in CM-associated parasites included other members of *Plasmodium* exported proteins such as *Pfmsp2, Pfmsp11* as well as other genes [PF11_0442, PF13_0090, PFC0185w, PF11_0381] encoding transcription factors with AP2 domain, ADP-ribosylation factor, membrane skeletal protein IMC1, respectively. PF10_0343 coding for S antigen was the most repressed transcript in CM-and AM-associated parasites. This gene might not be transcribed in these isolates.

### Confirmation of array results using quantitative rt-PCR

**Figure 2:**
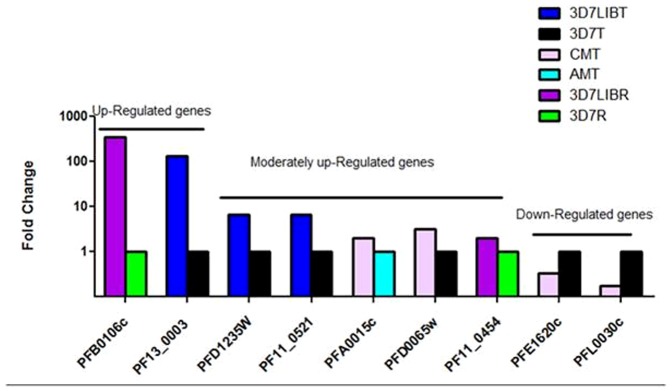
The expression level measured by quantitative rt-qPCR for arrays data confirmation. *PFB0106c* and *PF13_0003* transcripts were highly up-regulated (fold change> 6) in arrays of 3D7-Lib-Ring (R) versus 3D7-R and 3D7-Lib late trophozoïte (LT) versus 3D7-LT, respectively. *PFD1235W*, *PF11_0521*, *PFA0015c*, *PFD0065w*, and *PF11_0454* transcripts were moderately up-regulated (fold change range [2.5-5]) in 3D7-LT versus 3D7Lib-LT, CM-LT versus AM-LT, CM-LT versus 3D7-LT, and 3D7-Lib-R versus 3D7-R, respectively. PFE1620C and PFL0030C transcripts were down-regulated (fold change range [4 to 0]) in CM-LT versus 3D7-LT.

**Figure 3:**
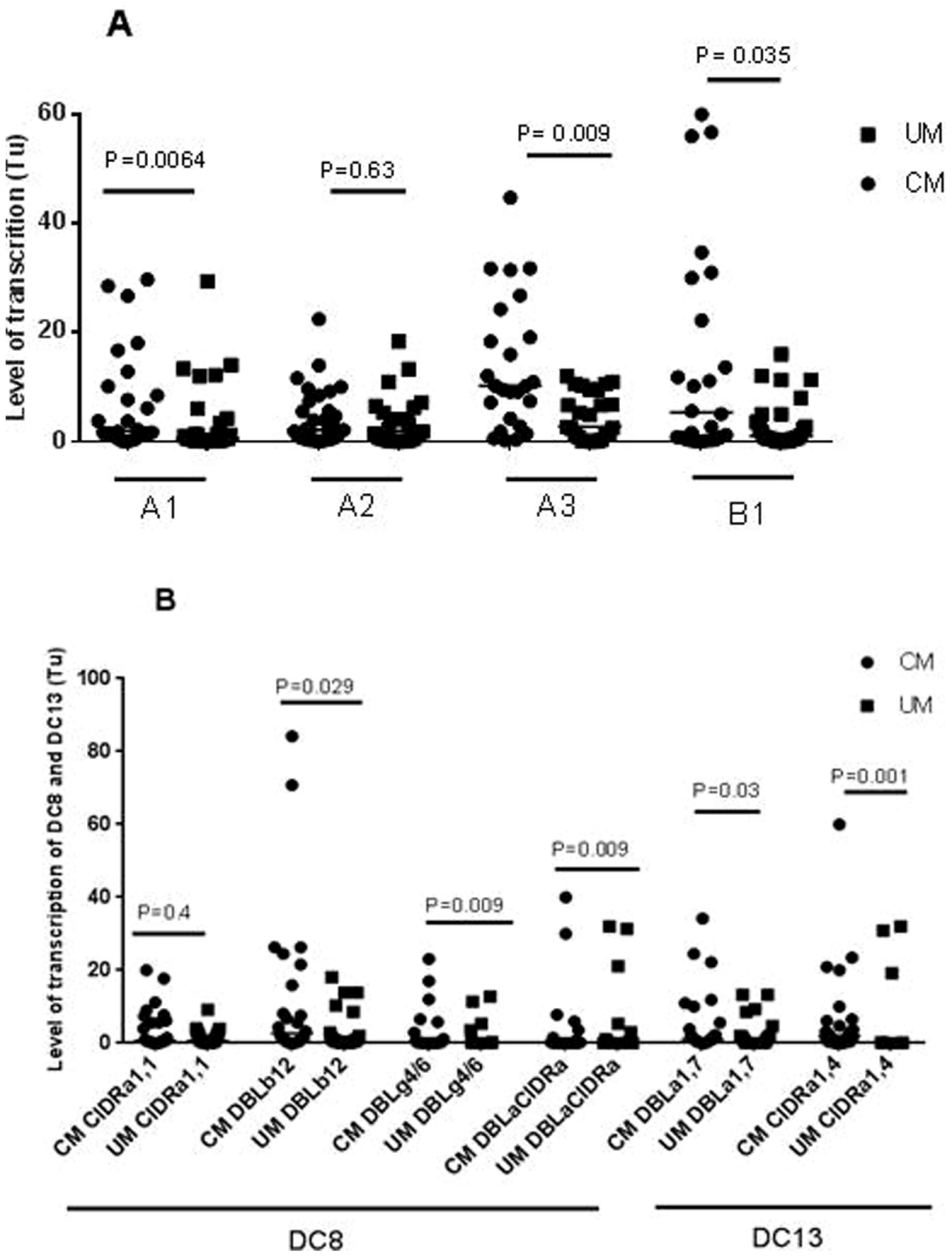
(A), transcription level of UPs A1, A3, B1, and (B), transcription level of DC 8, DC13 *var* genes in parasites from cerebral malaria (CM) and uncomplicated malaria (UM). The differential in transcription was compared between clinical groups by the Mann-Whitney U test. *P*-values are shown for each primer set.

The differential transcription level obtained by DNA hybridization arrays was further confirmed by qPCR using cDNA from the samples used for array hybridization (Figure 2) and cDNA from a second set of samples (Figure 3). A highly significant correlation (rho  =  0.775; *P* <0.0011, Spearman's rank correlation) between array and qPCR results was found, indicating that both methodological approaches yielded similar results and identified the genes that were either down- or up- regulated with accuracy (Figure S2).

### Bioinformatics analysis of differentially expressed genes

GO term analysis of up-regulated genes in CM-associated parasites compared to AM-associated parasites, as well as ring and late trophozoite stages of 3D7-Lib compared to those of 3D7, revealed GO terms of pathogenesis as the most over represented term (Table 2). Other over represented GO terms included cytoadherence to microvasculature mediated by symbiont protein, regulation of cell adhesion, and rosetting.

**Table 2:** Gene ontology term of differentially expressed genes.

	Best GOs	P-value	FDR(Benjamini)	Gene Ontology term description
**CMT versus AMT up- regulated genes**	GO:0009405	5 × 10^−4^	0.046	Pathogenesis
	GO:0020013	9 × 10^−4^	0.046	modulation by symbiont of host erythrocyte aggregation
	GO:0020013	9 × 10^−4^	0.046	Rosetting
	GO:0030155	9 × 10^−4^	0.046	regulation of cell adhesion
	GO:0034117	9 × 10^−4^	0.046	homotypic cell-cell adhesion
**3D7Lib T vs 3D7 T up-regulated genes**	GO:0009405	2 × 10^−24^	6 × 10^−22^	Pathogenesis
	GO:0044406	1 × 10^−13^	2 × 10^−11^	adhesion to host
	GO:0022407	5 ×10^−13^	4 × 10^−11^	regulation of cell-cell adhesion
	GO:0034109	1 × 10^−13^	8 × 10^−11^	homotypic cell-cell adhesion
	GO:0051701	2 × 10^−12^	1 × 10^-10^	interaction with host
**3D7 Lib R vs 3D7 R up-regulated genes**	GO:0009405	2 × 10^−19^	1 × 10^−17^	Pathogenesis
	GO:0044403	3 × 10^−17^	3 × 10^−16^	symbiosis, encompassing mutualism through parasitism
	GO:0034109	3 × 10^−17^	3 × 10^−16^	homotypic cell-cell adhesion
	GO:0020013	4 × 10^−17^	4 × 10^−16^	Rosetting
	GO:0051704	5 × 10^−17^	5 × 10^−16^	multi-organism process

Five most significant GO terms for Gene Ontology term analysis of differentially expressed gene set in cerebral malaria isolates and 3D7-Lib compared to asymptomatic malaria isolates and 3D7 clone, respectively. The reported p-values are based on the “OddsRatio score”, a measure of gene-enrichment. Benjamini & Hochberg false discovery rate control procedure was applied to correct for multiple comparisons. CMT, cerebral malaria-associated parasites, trophozoite stage; AMT, asymptomatic carrier-associated parasites, trophozoite stage; 3D7-Lib T, trophozoite stage of 3D7-Lib line; 3D7 T, trophozoite stage of 3D7; 3D7-Lib R, ring stage of 3D7-Lib line; 3D7 R, ring stage of 3D7.

Clustering analysis performed for up-regulated genes according to their fold change exhibited a network of two clusters in up-regulated gene set of the three compared conditions, CM versus AM, 3D7-Lib T (trophozoite) versus 3D7 T, and 3D7-Lib R (rings) versus 3D7 R. For CM up-regulated genes, the first cluster contained three genes with PF3D7_0201700/PFD0085c; encoding acylcoA gene separated from PF3D7_1301200/PF13_0010 gene encoding GBPH2 and PF3D7_0701900/PF07_0004 encoding *Plasmodium* exported protein. Similarly, comparison of the first cluster of 3D7-Lib T versus 3D7 T contained PF10_0038; gene encoding *40S ribosomal protein (S20e)* separated from PF3D7_0935900/PFI1735c *REX1*, PF3D7_1301700/MAL13P1.61 *Plasmodium exported protein, (GEXP07)*, PF3D7_1001500/PF10_0019 early transcribed membrane protein 10.1 *(ETRAMP10)*. The first cluster of 3D7-Lib R up-regulated genes also contained four genes with KAHRP-encoding gene with the highest fold change, followed by three PfEMP1 encoding genes; *PF13_0003*, *PF11_0521* and *PFD1235w*.

In three conditions, the second cluster contained the remaining up-regulated genes within two sub-networks in which some of the connected genes were grouped according to their chromosomal location or sequence and functional similarities, as PF3D7_0201600/PFB0080c, encoding PHISTb, PF3D7_0201800/PFB0090c, encoding RESA- like protein with PHIST and DNAJ domains.

## Discussion

Microarray-based transcriptomic studies are powerful approaches that have been successfully applied to identify many virulence genes in bacterial and cancer cell systems [41], [42]. Due to the limited amount of RNA that can be obtained from clinical samples of *P. falciparum*-infected individuals, only a few studies have investigated whole transcriptomes of clinical isolates [43]–[47]. These studies have demonstrated substantial differences in gene expression patterns between clinical isolates and laboratory-adapted *P. falciparum* strains.

The present study aimed to compare and analyze the whole transcriptome of *P. falciparum* parasites isolated from patients with distinct clinical features, including asymptomatic carriage, acute uncomplicated malaria, and cerebral malaria, in order to gain new insights into the regulation of gene transcription underlying malaria pathology. Our results indicate noticeable variation in gene transcription pattern of parasite isolates compared to 3D7, as well as between the parent 3D7 and selected 3D7-Lib. The observed differential in gene transcription may result from the difference in the transcription level of conserved genes but may also reflect the transcription of other *vsa* genes that belong to the same group in the case of field isolates since several variants could be detected by the same probe. Therefore, more *vsa* genes were detected in field parasites compared to 3D7, which is consistent with other findings [43], [44].

The observed variation in gene transcription pattern between parasites isolated from children with CM and parasites isolated from asymptomatic children may be related to the parasite growth rate as well as host environmental factors, such as fever and inflammation that differ drastically between these two groups. Likewise, the absence of differentially expressed genes between CM and UM transcriptomes suggests similar transcription pattern since these parasite groups multiply efficiently in a similar host environment during malaria episodes characterized by fever which is absent in AM-associated parasites. This result was not in agreement with our rt-qPCR finding concerning the up-regulation of UPs A1, A2, A3, B1, DC8 and DC13 *var* genes in CM-associated parasites compared to UM-associated parasites using more isolates (Figure 3, A, B). Therefore, the similar transcriptomic pattern between CM and UM isolates may not include *var* genes and their cognate partners like *phist* genes which may be concomitantly transcribed [50]. To confirm the equivalence between two transcriptomes, more array experiments using a higher number of isolates are needed.

We then focused our attention on genes which showed the same regulation of transcription in both CM-associated parasites and 3D7-Lib compared to AM-isolates and 3D7, respectively. Among these genes, there were genes encoding conserved proteins, exported protein, and variant surface proteins as *phist*, *rifin*, and UPs A1 A3 and B1 *var*. The difference in the transcription profile of a set of these genes confirmed by rt-qPCR showing the up-regulation of specific *var* gene groups targeted by UPs A1, A2, A3, B1, var2, var3 and DC8, DC13 in CM-associated parasites, compared to AM– and UM-associated parasites, might be of biological significance (Figure 3). These assumptions are reinforced by our test for enrichment on gene ontology categories of up-regulated genes in 3D7-Lib and CM-associated parasites which yielded the over representation of GO terms related with pathogenesis, regulation of cell or cell-cell adhesion, rosetting, and interaction with host. We also compared our transcriptomic results to published data and found that the majority of transcripts that were not detected in our study in ring and trophozoite stages of 3D7 and 3D7-Lib were also absent in previous array data [17], [18], suggesting untranscribed ORFs in *in vitro* cultivated parasites. However, many transcripts that were absent from the 3D7 transcriptome were present in our transcriptomic data of field isolates and in the transcriptome of other clinical isolates [47], suggesting that these transcripts are not essential for asexual *in vitro* survival within the red blood cells. Among the undetected transcripts in ring and trophozoite stages in 3D7 clone, most were amplified gene families that encode PfEMP1, RIFIN, STEVOR, and PFMC2TM as well as unknown hypothetical proteins.

We also compared our transcriptomic data from ring and late trophozoite stages of 3D7, as well as from parasite isolates, to those of the corresponding stages of 3D7 obtained by RNA sequencing [40] and found a highly significant correlation (p  =  2.2e-16, Kendall test) between these data. These results demonstrate the robustness of the array hybridization method to provide consistent data that are comparable to RNA sequencing and reflect the conserved nature of basic biological processes, including general metabolism and cell cycle, in both *in vivo* and *in vitro* conditions. This high correlation is also consistent with the predominance of ring stage parasites in the peripheral blood of infected individuals and changes in the transcription pattern leading to the progression and development towards the late trophozoite stage.

We subsequently investigated the function of 99 up-regulated genes in parasites from children with CM compared to the parasites from children with AM, and found that 28% of up-regulated genes encode exported proteins, with the majority (67%) of them being VSA-encoding genes, such as *var*, *rif*, *phist*, and *surfin*. These genes are implicated in parasite sequestration and immune evasion. Interestingly, *var* group A/Ups A1, A2, A3, and/or UpsB1, [*PF3D7_1150400/PF11_0521*, *PF3D7_1300300/PF13_0003*, *PF3D7_0937600/PFI1820w*, *PF3D7_0632800/PFF1595c*, *PF3D7_0425800/PFD1235w*] as well as CD8 and DC13 were confirmed to be up-regulated in CM-associated isolates and in the 3D7-Lib. *3D7 PF3D7_1150400/PF11_0521* displays DBLα-1.7 and CIDR-α1.2, while *PF3D7_0400400/PFD0020c* displays CIDRα-1.1, DBLβ12, DBLγ6. These architectural domains characterize Domain Cassettes (DC) DC13 and DC8, respectively, which were shown to be transcribed at higher levels in parasites from children with severe malaria in Tanzania [31], [48], as compared to the parasites from children with uncomplicated malaria. Similarly, *PF3D7_1300300/PF13_0003*, *PF3D7_0425800/PFD1235w* contain DC5 which was associated with severe malaria in one study [31] and also with parasite binding to the human receptor PECAM1 [49]. Furthermore, transcription of *PF3D7_0400400/PFD0020c* and that of its orthologs IT4var19, HB3var3, and IT4var7 were also shown to be highly induced in parasite lines selected for binding to human brain endothelial cells (HBEC) [51], [52].

Rif genes PF3D7_0631800/PFF1545w, PF3D7_1041000/PF10_0403, PF3D7_1041000/PFC1100w PF3D7_1150300/PF11_00520, PF3D7_0425700/PFD1230c, PF3D7_1100300/PF11_0009, phist a, b, c PF3D7_1478000/PF14_0752, PF3D7_1253300/PFL2565w, PF3D7_0702100/MAL7P1.7, PF3D7_0936900/PFI1785w, PF3D7_0830600/MAL8P1.4, PF3D7_0219700/PFB0900c, and PF3D7_1300300/PF13_0003 Pfemp1 were also up-regulated in 3D7-Lib and/or in both 3D7-Lib and parasites from CM patients. The phist a PF3D7_1478000/PF14_0752 was previously reported to be up-regulated in parasite lines selected for binding to HBEC [51]. Recently, a PHIST protein was found to interact with an erythrocyte cytoskeleton protein [53] and another PHIST member was found to bind the ATS part of PFEMP-1 and co-migrate with PFEMP-1 to the erythrocyte surface. It was also found in the same study that two PHIST members bind PFEMP-1 variants with different affinity suggesting specific PHIST/PFEMP1 combinations [50]. The rif genes PF3D7_1150300/PF11_00520, PF3D7_1100300/PF11_0009, PF3D7_0425700/PFD1230c, PF3D7_1300400/PF13_0004, were concomitantly up-regulated with their neighboring var genes PF3D7_1150400/PF11_0521, PF3D7_1100200/PF11_0008, PF3D7_0425800/PFD1235w, PF3D7_1300300/PF13_0003 in 3D7-Lib and were also detected in CM-associated parasites. These genes may be under the control of the same promoter region.

Other genes, such as *PF3D7_1301200*/*PF13_0010*, *PF3D7_1401000/PF14_0010*, and *PF3D7_0501300*/*PFE0065w*, were also found to be highly up-regulated in parasites from children with CM. *PF3D7_1401000*/*PF14_0010* and *PF3D7_1301200*/*PF13_0010*, encode GBPH and GBPH2, respectively. These proteins belong to Maurer's cleft proteins [54], either transiently during their export to the infected erythrocyte membrane, like PfEMP1, RIFIN [54]-[57], and PfMC-2TM [58], or are constitutive proteins of the Maurer's cleft itself, as *Plasmodium* skeleton-binding protein 1 (PfSBP1) which is encoded by *PF3D7_0501300*/*PFE0065w* [55]. PfSBP1 was demonstrated to be implicated in PfEMP1 trafficking to the surface of erythrocytes [59]. *PF3D7_1478000/Pf 14_0752* encodes MAHRP1 (Phist a), a Maurer's cleft resident protein that is also essential for PFEMP1 trafficking to the surface of infected red blood cells [60], [61]. These two genes were transcribed at higher levels in field isolates, compared to 3D7. MAHRP1 was among the 15 most up-regulated genes in parasite lines selected to adhere to HBEC [51].

Among the regulated genes, some code for antigenic proteins, such as *P. falciparum* sporozoite surface threonine- and asparagine-rich protein (STARP) (*PF3D7_07023005/PF07_0006*, *P. falciparum* ring-infected erythrocyte surface antigen *PF3D7_1149200/PF11_0509*, ring-infected erythrocyte surface antigen (RESA). These genes were found to be up-regulated in clinical isolates, as compared to laboratory-adapted strains [62]. The genes implicated in the transcription process, including *PF3D7_0318200*/PFC0805w DNA-directed RNA polymerase II, *PF3D7_1433500/PF14_0316* putative DNA topoisomerase II, *PF3D7_1220100/PFL0970w PF3D7_1231600/PFL1525c* pre-mRNA splicing factors were transcribed at higher levels in CM-associated parasites, compared to AM-associated parasites and 3D7. *PF3D7_1220100/PFL0970w* was highly expressed in parasites from children and pregnant women [47]. However, the transcription factors with ApiAp2 domains *PF3D7_1439500/PF14_0374*, *PF3D7_1143100/PF11_0442* were slightly down-regulated in CM-and AM-associated parasites, compared to 3D7 which is consistent with the findings of Vignali *et al*. [47].

Several differentially expressed genes in the present study were similarly regulated in other studies [58], supporting the variable nature of expression pattern of these genes between field isolates and 3D7 clone. For instance, (REX1) ring-exported protein 1, PF3D7_0935900/PFI1735c, (REX2) ring-exported protein 2, PF3D7_0936000/PFI1740; (GIG) gametocytogenesis-implicated protein PF3D7_0935600/PFI1720w, (STARP) sporozoite threonine- and asparagine-rich protein PF3D7_0702300/PF07_0006; lysophospholipase, putative PF3D7_0702200/PF07_0005, conserved *Plasmodium* protein with unknown function PF3D7_0702400/PF07_0007, (Pfg27) gamete antigen 27/25 PF3D7_1302100/PF13_0011 were all up-regulated in AM-and CM-associated isolates analyzed in our study and were also among the top 20 up-regulated genes in clinical isolates in a previous study [59]. REX1, REX2 and GIG genes are located in close proximity in chromosome 9, while STARP and putative lysophospholipase, as well as a conserved *Plasmodium* gene are located within a close distance in chromosome 7, suggesting co-regulation of gene transcription due to their proximity. Similarly, when examining the topological location of all up-regulated genes in parasites from CM patients compared to those from AM carriers, three to 12 genes were located on the same chromosome at a distance ranging from 5 to 100 kb, a distance within the variation of expression can be controlled by a single promoter or the same epigenetic mechanism.

In conclusion, we identified a set of specifically up-regulated genes in CM-associated parasites. Many of these genes encode proteins of diverse functions including remodeling of host cell, protein exports, surface antigens proteins, and cytoadherence which are likely to take part in parasite survival in the human host and pathogenesis. Due to the hypervariability of variant amplified genes as *var*, *phist* and *rifin* families the accuracy of their up-regulation in CM parasites has to be confirmed by whole RNA sequencing methods. In addition, some discrepancies in the stages of *in vitro* matured parasites may be at the origin of gene differential transcription, as gene expression during intra-erythrocytic cycle of the parasite is tightly regulated.

The potential role of these genes in CM deserves further investigations to identify new targets for malaria control.

## Supporting Information

Figure S1 **Histogram of raw p-values obtained from limma for the comparison of cerebral malaria with asymptomatic malaria.**
(TIF)

Figure S2 **Spearman's rank correlation (r  =  0.775;** ***P*** **<0.001) between arrays hybridization and qPCR methods for gene expression level.**
(TIF)

Figure S3 **Correlation circle plot: the variables are represented through their projections onto the plane defined by the new components (score vectors).** The variables being assumed to be of unit variance, their projections are inside a circle of radius 1 centered at the origin of the circle. Strongly associated (or correlated) variables are projected in the same direction from the origin. The greater the distance from the origin, the stronger is the association. Two circumferences of radius 1 and 0.5 are plotted to reveal the correlation structure of the data. Variables from the Bozdech et al. 2003 data set (from 1 to 48 hr) are indicated in red. Panel A, variables from the ring (R) stage; Panel B, variables from late trophozoites (T). Our data are indicated in blue.
(TIF)

Table S1 **Sequence of primers used in the RT-qPCR.**
(DOCX)

Table S2 **List of undetected transcripts in 3D7 and 3D7-Lib at late trophozoite stage.**
(XLSX)

Table S3 **List of undetected transcripts in 3D7 and 3D7-Lib at ring stage.**
(XLSX)

Table S4 **List of undetected transcripts in CM and AM isolates at trophozoite stage.**
(XLSX)

Table S5 **List of up- and down-regulated genes in 3D7 and 3D7-Lib at ring and late trophozoite stages and in CM and AM isolates at late trophozoite stage.**
(XLSX)

## References

[1] Greenwood B, Marsh K, Snow R (1991) Why do some African children develop severe malaria? Parasitol Today 7:277–281. 15463389 10.1016/0169-4758(91)90096-7

[2] Snow RW, Guerra CA, Noor AM, Myint HY, Hay SI (2005) The global distribution of clinical episodes of *Plasmodium falciparum* malaria. Nature 434:214–217. 15759000 10.1038/nature03342 PMC3128492

[3] Murray CJ, Rosenfeld LC, Lim SS, Andrews KG, Foreman KJ, et al (2012) Global malaria mortality between 1980 and 2010: a systematic analysis. Lancet 379:413–431. 22305225 10.1016/S0140-6736(12)60034-8

[4] World Health Organization (2012) Summary and Key Points. World Health OrganizationGeneva.

[5] Newton CR, Taylor TE, Whitten RO (1998) Pathophysiology of fatal *falciparum* malaria in African children. Am J Trop Med Hyg 58:673–683. 9598460 10.4269/ajtmh.1998.58.673

[6] Sachs J, Malaney P (2002) The economic and social burden of malaria. Nature 415:680–685. 11832956 10.1038/415680a

[7] Olliaro P, Wells TN (2009) The global portfolio of new antimalarial medicines under development Clin Pharmacol Ther. 85:584–595. 10.1038/clpt.2009.51 19404247

[8] World Health Organization (2013) World Malaria Report.

[9] Pongponratn E, Riganti M, Punpoowong B, Aikawa M (1991) Microvascular sequestration of parasitized erythrocytes in human *falciparum* malaria: a pathological study. Am J Trop Med Hyg 44:168–175. 2012260 10.4269/ajtmh.1991.44.168

[10] Marsh K, Otoo L, Hayes RJ, Carson DC, Greenwood BM (1989) Antibodies to blood stage antigens of *Plasmodium falciparum* in rural Gambians and their relation to protection against infection. Trans R Soc Trop Med Hyg 83:293–303. 2694458 10.1016/0035-9203(89)90478-1

[11] Bull PC, Lowe BS, Kortok M, Molyneux CS, Newbold CI, et al (1998) Parasite antigens on the infected red cell surface are targets for naturally acquired immunity to malaria. Nat Med 4:358–360. 9500614 10.1038/nm0398-358 PMC3836255

[12] Dodoo D, Staalsoe T, Giha H, Kurtzhals JA, Akanmori BD, et al (2001) Antibodies to variant antigens on the surfaces of infected erythrocytes are associated with protection from malaria in Ghanaian children. Infect Immun 69:3713–3718. 11349035 10.1128/IAI.69.6.3713-3718.2001 PMC98376

[13] Mackintosh CL, Beeson JG, Marsh K (2004) Clinical features and pathogenesis of severe malaria. Trends Parasitol 20:597–603. 15522670 10.1016/j.pt.2004.09.006

[14] Ranjit MR, Das A, Das BP, Das BN, Dash BP, et al (2005) Distribution of *Plasmodium falciparum* genotypes in clinically mild and severe malaria cases in Orissa, India. Trans R Soc Trop Med Hyg 99:389–395. 15780346 10.1016/j.trstmh.2004.09.010

[15] Kwiatkowski DP (2005) How malaria has affected the human genome and what human genetics can teach us about malaria. Am J Hum Genet 77:171–192. 16001361 10.1086/432519 PMC1224522

[16] Gardner MJ, Hall N, Fung E, White O, Berriman M, et al (2002) Genome sequence of the human malaria parasite *Plasmodium falciparum* . Nature 419:498–511. 12368864 10.1038/nature01097 PMC3836256

[17] Le Roch KG, Zhou Y, Blair PL, Grainger M, Moch JK, et al (2003) Discovery of gene function by expression profiling of the malaria parasite life cycle. Science 301:1503–1508. 12893887 10.1126/science.1087025

[18] Bozdech Z, Llinas M, Pulliam BL, Wong ED, Zhu J, et al (2003) The transcriptome of the intraerythrocytic developmental cycle of *Plasmodium falciparum* . PLoS Biol 1:E5. 12929205 10.1371/journal.pbio.0000005 PMC176545

[19] Kuss C, Gan CS, Gunalan K, Bozdech Z, Sze SK, et al. (2012) Quantitative proteomics reveals new insights into erythrocyte invasion by Plasmodium falciparum. Mol Cell Proteomics11M111 010645. 10.1074/mcp.M111.010645 PMC3277754 22023809

[20] Patra KP, Johnson JR, Cantin GT, Yates JR III, Vinetz JM (2008) Proteomic analysis of zygote and ookinete stages of the avian malaria parasite *Plasmodium gallinaceum* delineates the homologous proteomes of the lethal human malaria parasite *Plasmodium falciparum* . Proteomics 8:2492–2499. 18563747 10.1002/pmic.200700727 PMC2637033

[21] Staalsoe T, Nielsen MA, Vestergaard LS, Jensen AT, Theander TG, et al (2003) In vitro selection of *Plasmodium falciparum* 3D7 for expression of variant surface antigens associated with severe malaria in African children. Parasite Immunol 25:421–427. 14651589 10.1111/j.1365-3024.2003.00652.x

[22] Wang CW, Magistrado PA, Nielsen MA, Theander TG, Lavstsen T (2009) Preferential transcription of conserved rif genes in two phenotypically distinct *Plasmodium falciparum* parasite lines. Int J Parasitol 39:655–664. 19162031 10.1016/j.ijpara.2008.11.014

[23] Trager W, Jensen JB (1976) Human malaria parasites in continuous culture. Science 193:673–675. 781840 10.1126/science.781840

[24] Jensen JB (1978) Concentration from continuous culture of erythrocytes infected with trophozoites and schizonts of *Plasmodium falciparum* . Am J Trop Med Hyg 27:1274–1276. 365006 10.4269/ajtmh.1978.27.1274

[25] Kirchgatter K, Portillo Hdel A (2002) Association of severe noncerebral *Plasmodium falciparum* malaria in Brazil with expressed PfEMP1 DBL1 alpha sequences lacking cysteine residues. Mol Med 8:16–23. 11984002 PMC2039937

[26] Bull PC, Pain A, Ndungu FM, Kinyanjui SM, Roberts DJ, et al (2005) *Plasmodium falciparum* antigenic variation: relationships between in vivo selection, acquired antibody response, and disease severity. J Infect Dis 192:1119–1126. 16107968 10.1086/432761

[27] Kyriacou HM, Stone GN, Challis RJ, Raza A, Lyke KE, et al (2006) Differential var gene transcription in *Plasmodium falciparum* isolates from patients with cerebral malaria compared to hyperparasitaemia. Mol Biochem Parasitol 150:211–218. 16996149 10.1016/j.molbiopara.2006.08.005 PMC2176080

[28] Rouillard JM, Zuker M, Gulari E (2003) OligoArray 2.0: design of oligonucleotide probes for DNA microarrays using a thermodynamic approach. Nucleic Acids Res 31:3057–3062. 12799432 10.1093/nar/gkg426 PMC162330

[29] Larkin MA, Blackshields G, Brown NP, Chenna R, McGettigan PA, et al (2007) Clustal W and Clustal X version 2.0. Bioinformatics 23:2947–2948. 17846036 10.1093/bioinformatics/btm404

[30] Hooper LV, Wong MH, Thelin A, Hansson L, Falk PG, et al (2001) Molecular analysis of commensal host-microbial relationships in the intestine. Science 291:881–884. 11157169 10.1126/science.291.5505.881

[31] Lavstsen T, Turner L, Saguti F, Magistrado P, Rask TS, et al (2012) *Plasmodium falciparum* erythrocyte membrane protein 1 domain cassettes 8 and 13 are associated with severe malaria in children. Proc Natl Acad Sci U S A 109:E1791–1800. 22619319 10.1073/pnas.1120455109 PMC3387094

[32] Gentleman RC, Carey VJ, Bates DM, Bolstad B, Dettling M, et al (2004) Bioconductor: open software development for computational biology and bioinformatics. Genome Biol 5:R80. 15461798 10.1186/gb-2004-5-10-r80 PMC545600

[33] Smyth GK, Michaud J, Scott HS (2005) Use of within-array replicate spots for assessing differential expression in microarray experiments. Bioinformatics 21:2067–2075. 15657102 10.1093/bioinformatics/bti270

[34] Bolstad BM, Irizarry RA, Astrand M, Speed TP (2003) A comparison of normalization methods for high density oligonucleotide array data based on bias and variance. Bioinformatics 19:185–193. 12538238 10.1093/bioinformatics/19.2.185

[35] Smyth G (2004) Linear models and empirical Bayes methods for assessing differential expression in microarray experiments. Statistical Applications in Genetics and Molecular Biology 3. 10.2202/1544-6115.1027 16646809

[36] Benjamini Y, Drai D, Elmer G, Kafkafi N, Golani I (2001) Controlling the false discovery rate in behavior genetics research. Behav Brain Res 125:279–284. 11682119 10.1016/s0166-4328(01)00297-2

[37] Falcon S, Gentleman R (2007) Using GOstats to test gene lists for GO term association. Bioinformatics 23:257–258. 17098774 10.1093/bioinformatics/btl567

[38] Lambert ZV, Durand RM (1975) Some precautions in using canonical analysis. J Marketing Res 12:468–475 doi: 10.2307/3151100

[39] González I, Déjean S, Martin PGP, Baccini A (2008) CCA: An R package to extend canonical correlation analysis. J StatSoftware 23:1–14.

[40] Bartfai R, Hoeijmakers WA, Salcedo-Amaya AM, Smits AH, Janssen-Megens E, et al (2010) H2A.Z demarcates intergenic regions of the *Plasmodium falciparum* epigenome that are dynamically marked by H3K9ac and H3K4me3. PLoS Pathog 6:e1001223. 21187892 10.1371/journal.ppat.1001223 PMC3002978

[41] Mahan MJ, Slauch JM, Mekalanos JJ (1993) Selection of bacterial virulence genes that are specifically induced in host tissues. Science 259:686–688. 8430319 10.1126/science.8430319

[42] Alizadeh AA (2000) Distinct types of diffuse large B-cell lymphoma identified by gene expression profiling. Nature 403:503–511. 10676951 10.1038/35000501

[43] Daily JP, Le Roch KG, Sarr O, Fang X, Zhou Y, et al (2004) In vivo transcriptional profiling of *Plasmodium falciparum* . Malar J 3:30. 15296511 10.1186/1475-2875-3-30 PMC514566

[44] Daily JP, Le Roch KG, Sarr O, Ndiaye D, Lukens A, et al (2005) In vivo transcriptome of *Plasmodium falciparum* reveals overexpression of transcripts that encode surface proteins. J Infect Dis 191:1196–1203. 15747257 10.1086/428289 PMC2582152

[45] Daily JP, Scanfeld D, Pochet N, Le Roch K, Plouffe D, et al (2007) Distinct physiological states of *Plasmodium falciparum* in malaria-infected patients. Nature 450:1091–1095. 18046333 10.1038/nature06311

[46] Tuikue Ndam N, Bischoff E, Proux C, Lavstsen T, Salanti A, et al (2008) *Plasmodium falciparum* transcriptome analysis reveals pregnancy malaria associated gene expression. PLoS One 3:e1855. 18365010 10.1371/journal.pone.0001855 PMC2267001

[47] Vignali M, Armour CD, Chen J, Morrison R, Castle JC, et al (2011) NSR-seq transcriptional profiling enables identification of a gene signature of *Plasmodium falciparum* parasites infecting children. J Clin Invest 121:1119–1129. 21317536 10.1172/JCI43457 PMC3046638

[48] Bertin G, Lavstsen T, Guillonneau F, Doritchamou J, Wang CW, et al (2013) Expression of the domain cassette 8 *Plasmodium falciparum* erythrocyte membrane protein 1 is associated with cerebral malaria in Benin. PLoS One8:e68368. 10.1371/journal.pone.0068368 PMC3726661 23922654

[49] Turner L, Lavstsen T, Berger SS, Wang CW, Petersen JE, et al (2013) Severe malaria is associated with parasite binding to endothelial protein C receptor. Nature 498:502–505. 23739325 10.1038/nature12216 PMC3870021

[50] Oberli A, Slater L, Brand F, Munwiler-Paclakto E, Rush S, et al (2014) APHIST protein interact with the intracellular ATS domain of PfEMP1, localizes to knobs and co-migrates with PfEMP1. Poster presentation 10^th^ annual BioMalPar.

[51] Claessens A, Adams Y, Ghumra A, Lindergard G, Buchan CC, et al (2012) A subset of group A-like var genes encodes the malaria parasite ligands for binding to human brain endothelial cells. Proc Natl Acad Sci U S A 109:E1772–1781. 22619330 10.1073/pnas.1120461109 PMC3387129

[52] Avril M, Tripathi AK, Brazier AJ, Andisi C, Janes JH, et al (2012) A restricted subset of *var* genes mediates adherence of *Plasmodium falciparum*-infected erythrocytes to brain endothelial cells. Proc Natl Acad Sci U S A 109:E1782–1790. 22619321 10.1073/pnas.1120534109 PMC3387091

[53] Parish LA, Mai DW, Jones ML, Kitson EL, Rayner JC (2013) A member of the *Plasmodium falciparum* PHIST family binds to the erythrocyte cytoskeleton component band 4.1, Malar J. 12:160. 10.1186/1475-2875-12-160 PMC3658886 23663475

[54] Lanzer M, Wickert H, Krohne G, Vincensini L, Braun-Breton C (2006) Maurer's clefts: a novel multi-functional organelle in the cytoplasm of *Plasmodium falciparum*-infected erythrocytes. Int J Parasitol 36:23–36. 16337634 10.1016/j.ijpara.2005.10.001

[55] Petter M, Haeggstrom M, Khattab A, Fernandez V, Klinkert MQ, et al (2007) Variant proteins of the *Plasmodium falciparum* RIFIN family show distinct subcellular localization and developmental expression patterns. Mol Biochem Parasitol 156:51–61. 17719658 10.1016/j.molbiopara.2007.07.011

[56] Winter G, Kawai S, Haeggstrom M, Kaneko O, von Euler A, et al (2005) SURFIN is a polymorphic antigen expressed on *Plasmodium falciparum* merozoites and infected erythrocytes. J Exp Med 201:1853–1863. 15939796 10.1084/jem.20041392 PMC2213267

[57] Kaviratne M, Khan SM, Jarra W, Preiser PR (2002) Small variant STEVOR antigen is uniquely located within Maurer's clefts in *Plasmodium falciparum*-infected red blood cells. Eukar Cell 1:926–935. 10.1128/EC.1.6.926-935.2002 PMC138759 12477793

[58] Sam-Yellowe TY, Florens L, Johnson JR, Wang T, Drazba JA, et al (2004) A *Plasmodium* gene family encoding Maurer's cleft membrane proteins: structural properties and expression profiling. Genome Res 14:1052–1059. 15140830 10.1101/gr.2126104 PMC419783

[59] Maier AG, Rug M, O'Neill MT, Beeson JG, Marti M, et al (2007) Skeleton-binding protein 1 functions at the parasitophorous vacuole membrane to traffic PfEMP1 to the *Plasmodium falciparum*-infected erythrocyte surface. Blood 109:1289–1297. 17023587 10.1182/blood-2006-08-043364 PMC1785152

[60] Spycher C, Rug M, Klonis N, Ferguson DJ, Cowman AF, et al (2006) Genesis of and trafficking to the Maurer's clefts of *Plasmodium falciparum*-infected erythrocytes. Mol Cell Biol 26:4074–4085. 16705161 10.1128/MCB.00095-06 PMC1489082

[61] Spycher C, Rug M, Pachlatko E, Hanssen E, Ferguson D, et al (2008) The Maurer's cleft protein MAHRP1 is essential for trafficking of PfEMP1 to the surface of *Plasmodium falciparum*-infected erythrocytes. Mol Microbiol 68:1300–1314. 18410498 10.1111/j.1365-2958.2008.06235.x

[62] Mackinnon MJ, Li J, Mok S, Kortok MM, Marsh K, et al (2009) Comparative transcriptional and genomic analysis of *Plasmodium falciparum* field isolates. PLoS Pathog 5:e1000644. 19898609 10.1371/journal.ppat.1000644 PMC2764095

