# Supplementary figures and images for: Differences in Gene Transcriptomic Pattern of Plasmodium falciparum in Children with Cerebral Malaria and Asymptomatic Carriers
Source: PLoS One. 2014 Dec 5;9(12):e114401. doi: 10.1371/journal.pone.0114401 (PMC4257676; doi:10.1371/journal.pone.0114401)

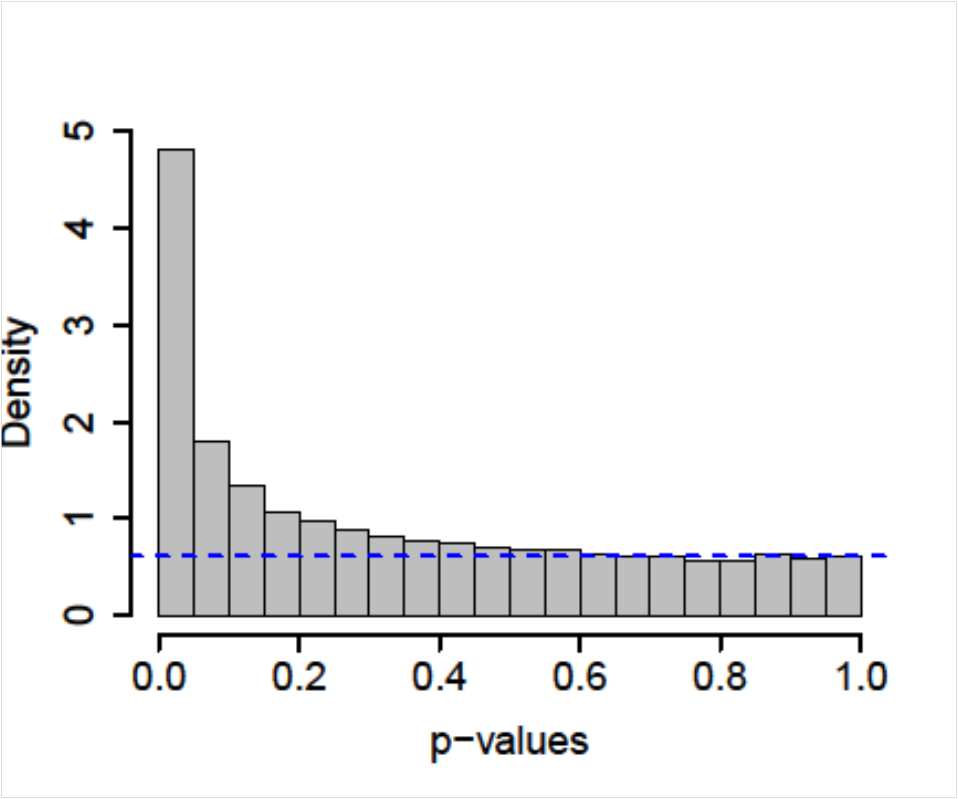

Supplement: Figure S1 — Histogram of raw p-values obtained from limma for the comparison of cerebral malaria with asymptomatic malaria. (TIF) [file pone.0114401.s001.tif]

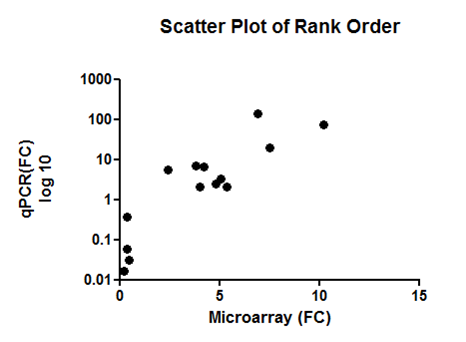

Supplement: Figure S2 — Spearman's rank correlation (r = 0.775; P <0.001) between arrays hybridization and qPCR methods for gene expression level. (TIF) [file pone.0114401.s002.tif]

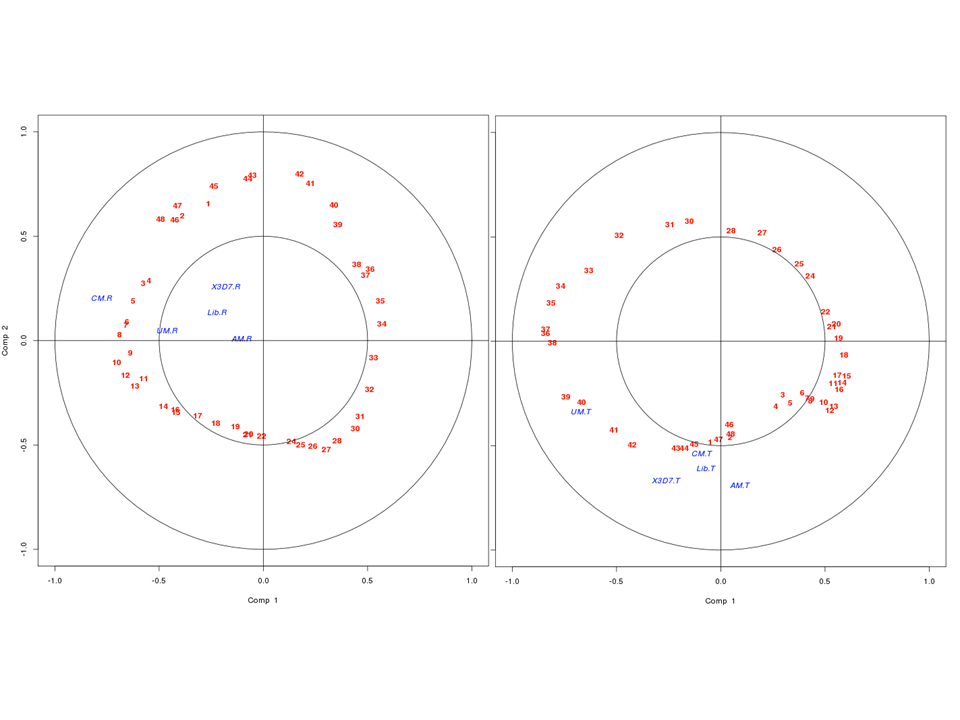

Supplement: Figure S3 — Correlation circle plot: the variables are represented through their projections onto the plane defined by the new components (score vectors). The variables being assumed to be of unit variance, their projections are inside a circle of radius 1 centered at the origin of the circle. Strongly associated (or correlated) variables are projected in the same direction from the origin. The greater the distance from the origin, the stronger is the association. Two circumferences of radius 1 and 0.5 are plotted to reveal the correlation structure of the data. Variables from the Bozdech et al. 2003 data set (from 1 to 48 hr) are indicated in red. Panel A, variables from the ring (R) stage; Panel B, variables from late trophozoites (T). Our data are indicated in blue. (TIF) [file pone.0114401.s003.tif]
