# Supplementary material for: Differences in Gene Transcriptomic Pattern of Plasmodium falciparum in Children with Cerebral Malaria and Asymptomatic Carriers
Source: PLoS One. 2014 Dec 5;9(12):e114401. doi: 10.1371/journal.pone.0114401 (PMC4257676; doi:10.1371/journal.pone.0114401)
Supplement: Table S1 — Sequence of primers used in the RT-qPCR. (DOCX) [file pone.0114401.s004.docx]

Liste of used primers

|  | Forword primers | Reverse primers |
| --- | --- | --- |
| PF3D7_0202200 /PFB0106c | CGTTCCTCCATTTATGGTTTAT | GTGCTTCATTTCTGCGTTGT |
| PF3D7_1300300 /PF13_0003 | CACAGGTATGGGAAGCAATG | CCATACAGCCGTGACTGTTC |
| PF3D7_0425800 /PFD1235w | AAACACGTTGAATGGCGATA | GACGCCGAGGAGGTAAATAG |
| PF3D7_1144000 /PF11_0454 | CAGGAAGTGCTCAGCTACAT | CTGACATGACCAGAGATAGC |
| PF3D7_1150400 /PF11_0521 | TGCTGAAGACCAAATTGAGC | TGCTGAAGACCAAATTGAGC |
| PF3D7_0401500 /PFD0065w | CCCGTGGTATATGTTCTTGC | GGTTGCTATACTGGCAGTAA |
| PF3D7_0100300 /PFA0015c | CGTAAAACATGGTGGGATGA | GGCCCATTCAGTTAACCATC |
| PF3D7_1200600 /PFL0030c | TGGTGATGGTACTGCTGGAT | TTTATTTTCGGCAGCATTTG |
| PF3D7_0532700 /PFE1620c | GATAGTGGTCGTGAAGAGGT | CGTTTGAGCATCTCATCATG |
| A1 | TTGGGRAATBTGTTAGTTAYRGCAA | CTGCAAAACTKCGWGCAAG |
| A2 | GTTCCAASGATCCATTRGATGTATTA | AACCCATCTGTRRATGATATACCTATGGA |
| A3 | AGGTAATGTTTTAGATGATGGTAT | ACCAGAATATACATTATTTGATACATA |
| B1 | CATCCGCCATGCAAGTATAA | CGTGCACGATTTCGATTTTT |
| CIDRα1.1 | TAARGTGGGAACATCAACTTAAGAAT | CTAAATCTTYCGTAAATTGATCCCAT |
| DBLb12 & DBLb3/5 | CATCAAGTKTGGAGAGCTATGAAATGTG | TAATCTTCTATKGGGATACCATTACA |
| DBLg4/6 of DC8 | AGAAACAHATTTTGCKTGG | CCATGTTCWTTCCACCAGTCDTCAAG |
| CIDRα 1.1 | TAARGTGGGAACATCAACTTAAGAAT | CTAAATCTTYCGTAAATTGATCCCAT |
| DBLγ4/6, | AGAAACAHATTTTGCKTGG | CCATGTTCWTTCCACCAGTCDTCAAG |
| DBLa1.7 | GATTAYGTCCCTCAATTTTTAMGWTGGT | GTTTACGAAATGCTTCTCGTTGATTRCC |
| CIDRα1.4 | GGGTTAGAAAWTTMTTAATAGACACTA | CTTTTTGTTTAACCCATYTGTCAAAAC |
